# Therapeutic application of mesenchymal stem cell-derived exosomes in skin wound healing

**DOI:** 10.3389/fbioe.2024.1428793

**Published:** 2024-08-05

**Authors:** Yunhan Sun, Shun Zhang, Yukai Shen, Haoyang Lu, Xincan Zhao, Xin Wang, Yongkai Wang, Taiping Wang, Bing Liu, Lan Yao, Jie Wen

**Affiliations:** ^1^ School of Stomatology, Shandong First Medical University and Shandong Academy of Medical Sciences, Jinan, Shandong, China; ^2^ Eye Hospital of Shandong First Medical University, Jinan, Shandong, China

**Keywords:** skin wound healing, exosoems, bioengeneering, hydrogel, nanofiber

## Abstract

Wound healing is a complicated obstacle, especially for chronic wounds. Mesenchymal stem cell-derived exosomes may be a promising cell-free approach for treating skin wound healing. Exosomes can accelerate wound healing by attenuating inflammation, promoting angiogenesis, cell proliferation, extracellular matrix production and remodeling. However, many issues, such as off-target effects and high degradation of exosomes in wound sites need to be addressed before applying into clinical therapy. Therefore, the bioengineering technology has been introduced to modify exosomes with greater stability and specific therapeutic property. To prolong the function time and the local concentration of exosomes in the wound bed, the use of biomaterials to load exosomes emerges as a promising strategy. In this review, we summarize the biogenesis and characteristics of exosomes, the role of exosomes in wound healing, and the therapeutic applications of modified-exosomes in wound healing. The challenges and prospects of exosomes in wound healing are also discussed.

## 1 Introduction

Wound healing is a complicated process, especially for chronic wounds, such as diabetic ulcers. Prolonged wound healing is an intractable problem for clinicians and a heavy burden for patients and society. Therefore, many different therapies have been introduced in wound care, including skin flap transplantation, skin substitutes, which methods are limited by high cost, infection, scarring during cutaneous wound healing. As translational and regenerative medicine progress, stem cell-based therapy have gained wide popularity due to their regenerative capacity (Joseph et al., 2020). Corroborating evidence shows that stem cell administration promotes regenerative healing of wounded skin primarily mainly through paracrine mechanisms by releasing growth factors and extracellular vesicles (EVs), including exosomes (Tkach and Théry, 2016; Zhang et al., 2020). The contents of EVs from stem cells were transferred to surrounding microenvironment and exhibit their therapeutic effects even when parental cells were cleared by the host. Moreover, EVs are easily and stably stored, not rejected by the immune system, and have a homing effect. Therefore, the use of extracellular vesicles presents a potential cell-free translational therapy (Ho et al., 2023).

Exosomes are a subset of small lipid bilayer EVs, with 40–160 nm in diameter. They are secreted from most cells into extracellular fluids by multivesicular bodies (MVBs), including stem cells and immune cells involved in wound healing (An et al., 2021). Exosomes are important participants in intercellular communication by transferring their cargo contents (proteins, RNA, lipids and other biologically active components) to the cytoplasm of target cells, thereby changing the biological properties of the recipient cells. Accumulated evidences verifies that exosomes can inhibit inflammation, enhance neovascular formation, promote cell proliferation and stimulate collage deposition to accelerate wound healing (Ha et al., 2020; Zhou et al., 2020; An et al., 2021). There are increasing studies describing a relationship between exosomes and cutaneous wound healing and summarized their underlying molecular mechanisms.

Although exosomes have great potential for accelerating skin wound healing, how to maximize the therapeutic effect of exosomes depends on other factors, like exosomes engineering and the delivery methods of exosomes (Dong et al., 2023; Zhao et al., 2023). In this review, we first described the characteristic of exosomes, the role of exosomes in wound healing process. More importantly, we summarized and discussed the application of exosomes to the wound healing, including how to modify exosomes by bioengineering and the delivery methods of exosomes in detail.

## 2 Biogenesis and characteristic of exosomes

**FIGURE 1:**
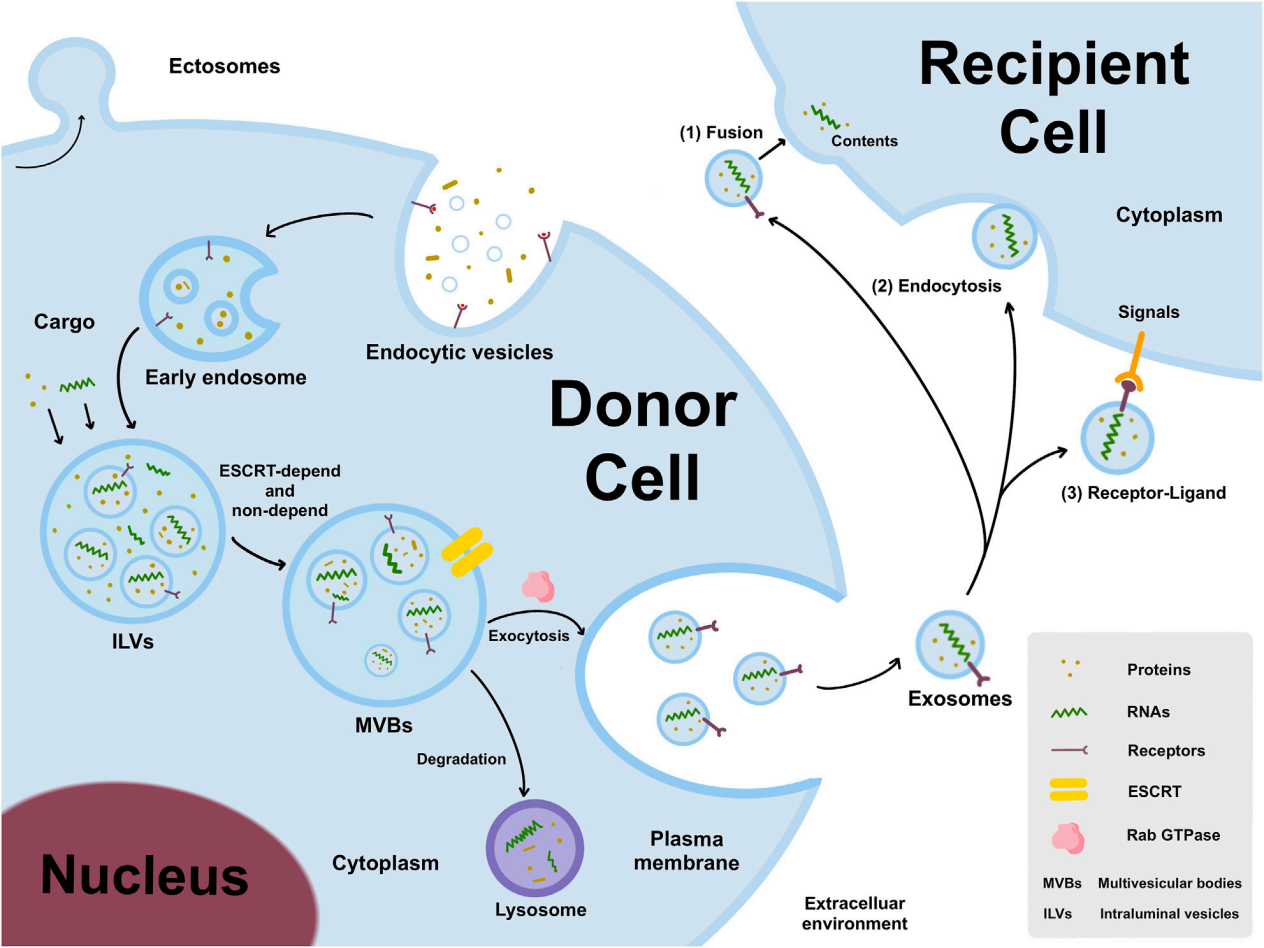
The generation and release of exosomes. Early endosomes are formed by an endosomal way, matured to form MVBs by ESCRT-depend and non-depend pathways, and then secreted into intercellular microenvironment. Exosomes can transmit donor cell information to recipient cells through three different pathways, including 1) exosomes directly fuse with cell membranes through interacting with trans-membrane proteins on the surface of recipient cells; 2) exosomes recruit into the cell body through filopodia and endocytosed by recipient cells; 3) ligands on exosomes interact with receptors on the membrane of recipient cells, and then trigger intracellular signaling cascades.

The EVs are generally classified into two major categories: ectosomes and exosomes. Ectosomes are generated by directly budding outwards from the plasma membrane, producing large vesicles with the size range of 50 nm to 1 um in diameter. By contrast, exosomes are formed by a endosomal way, with 40nm–160 nm in diameter (Meldolesi, 2018). The generation of exosomes is a tightly regulated process, mainly including three main processes (Figure 1): endocytic vesicles, multivesicular bodies (MVBs) formation, and secretion of exosomes (Arya et al., 2024).

The cell membrane buds inward to form early endosomes, in which “intraluminal vesicles” (ILVs) develop, and then matured to form MVBs. Generally, the mechanism that described the generation of ILVs and MVBs is “Endosome Sorting Complex Required for Transport” (ESCRT) -dependent and non-dependent pathways (Arya et al., 2024). The Rab family of small GTPases plays a vital regulatory mediator in intracellular transport and secretion. Besides, Rab27a and Rab27b are demonstrated to promote the formation and stability of MVBs (Ruivo et al., 2022). KIBRA was shown to regulate exosome secretion by blocking proteasome degradation of Rab27a, and KIBRA depletion may result in an increase in the quantity and size of MVBs (Song et al., 2019). Generally, the MVBs transfer to the plasma membrane and released into the extracellular space, but some are degraded by fusing with the lysosome (Arya et al., 2024).

Exosomes, often known as “cargo,” usually encapsulate variety of bioactive molecules depending on the source and state of cell and its microenvironment. Exosomes can transfer the effector molecules to the recipient cells via following three pathways (Figure 1): I) Exosomes directly deliver inclusions to recipient cells by combining with recipient cells by fusing with the cell membrane through interacting with trans-membrane proteins on the surface; II) Exosomes are recruited to cell body by filopodia and then endocytosed by recipient cells that resemble virus entry to cells; III) The ligands on exosomes interact with the receptor on recipient cell membrane, and then trigger the intracellular signaling cascade (Liang et al., 2021).

Exosomes involved in skin wound healing are mainly derived from adipose-derived stem cells (ADSCs) (Jiang et al., 2022a; Liang et al., 2022; Shi et al., 2022), bone marrow mesenchymal stem cells (BMSCs) (Pomatto et al., 2021; Born et al., 2022; Wang et al., 2022), umbilical cord-derived mesenchymal stem cells (UCMSCs) (Yang et al., 2020; Yan et al., 2022b), macrophages (Li et al., 2019), human umbilical vein endothelial cells (HUVECs), epidermal stem cells (ESCs) (Xu et al., 2020) and dermal fibroblasts (Han et al., 2021). Based on the abundant sources and assured efficacy, ADSCs and BMSCs were the most dependable source of exosomes, which are studied by most of researches. Besides, exosomes are also isolated from non-cellular sources, such as serum (Xu et al., 2018; Chen L. et al., 2021), platelet-rich plasma (PRP) (Guo et al., 2017) and milk (Yan et al., 2022a). Although they have abundant sources, their stability and effectiveness still need to be further confirmed.

Different approaches have been developed for the isolation and purification of exosomes, including ultracentrifugation, ultrafiltration, precipitation, size exclusion chromatography, immunoaffinity capture and microfluidics (Coumans et al., 2017; Li et al., 2017). To date, the widely accepted method is the differential ultracentrifugation. No uniform strategy has been established for exosome isolation with the highest quality and properties, and a combination of different methods is usually required to achieve optimal results. The isolation of exosomes can be detected by transmission electron microscope (TEM) and nanoparticle tracking analysis (NTA), and the exosomal markers, including tetraspanins (CD9, CD81, and CD63) (Fan et al., 2023; Ai et al., 2024), tumor susceptibility gene 101 proteins (TSG101) (Liu et al., 2021), heat shock proteins (HSP60 and HSP90) (Liu et al., 2021; Ghafourian et al., 2022) can be analyzed by Western blotting method.

## 3 Wound healing process

**FIGURE 2:**
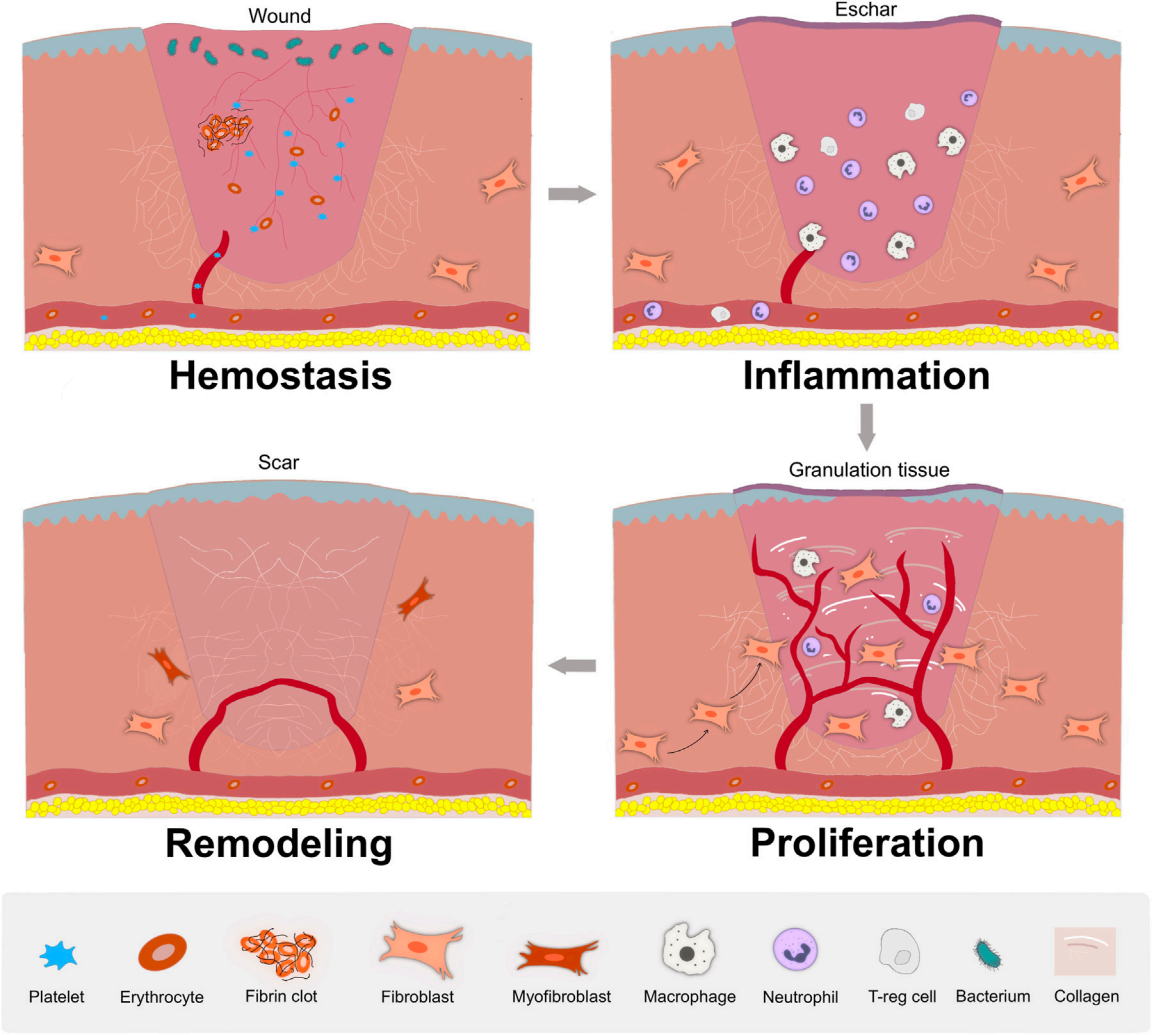
The stages of wound healing. In hemostasis phase, the platelet aggregates to the wound to block blood loss and an initial fibrin clot is formed; In inflammatory phase, local and system immune respond to attenuate invading bacteria and pathogens, with the recruitment and activation of neutrophils and macrophages; In proliferative phase, granulation tissue is formed, along with abundant blood vessels, re-epithelialization, fibroblasts proliferation and migration, and extracellular matrix synthesis; In remodeling phase, with matrix deposition and remodeling, the wound entirely closed, with possible scar formation.

Wound healing proceeds through four distinct but overlapping phases (Figure 2): Hemostasis phase, inflammation response, cellular proliferation and tissue remodeling phase (Wilkinson and Hardman, 2020; Bian et al., 2022). i) Hemostasis phase: the primary step of healing to end bleeding. The platelets are activated and support a stable clot by thrombin, contributing to the generation of a fibrin clot entrapping blood cells in the injured area (Hoffman, 2018; Zhu et al., 2018). ii) Inflammatory phase: deteriorating bacteria and pathogens, local and system immune response. Above events mainly depend on the recruitment and activation of neutrophils and macrophages which clear invading bacteria and cellular debris via direct functions or the release of multiple mediators (Shah and Amini-Nik, 2017; Raziyeva et al., 2021). iii) Proliferative phase: granulation tissue is formed, which depends on epithelialization and myofibroblasts functions. Epithelial cells proliferate and migrate from the margins to the injured area to form epithelialization (Ben Amar and Wu, 2014). Fibroblasts proliferate and synthesize matrix and collagen to provide an external bed for cell attachment scaffolding and repair (Shpichka et al., 2019). Meanwhile, abundant blood vessels is urgently required to provide sufficient oxygen and nutrients for granulation tissue (Wilkinson and Hardman, 2020). (iiii) Remodeling phase: collagen deposition and the entirely closed wound. Collagen is cross-linked between collagen fibers and remodeled gradually from type III to type I collagen. New tissue completely covers the injured area, and slowly attains strength and flexibility, and finally scar tissue is formed (Shpichka et al., 2019; Wilkinson and Hardman, 2020).

## 4 The role of exosome in wound healing process

**FIGURE 3:**
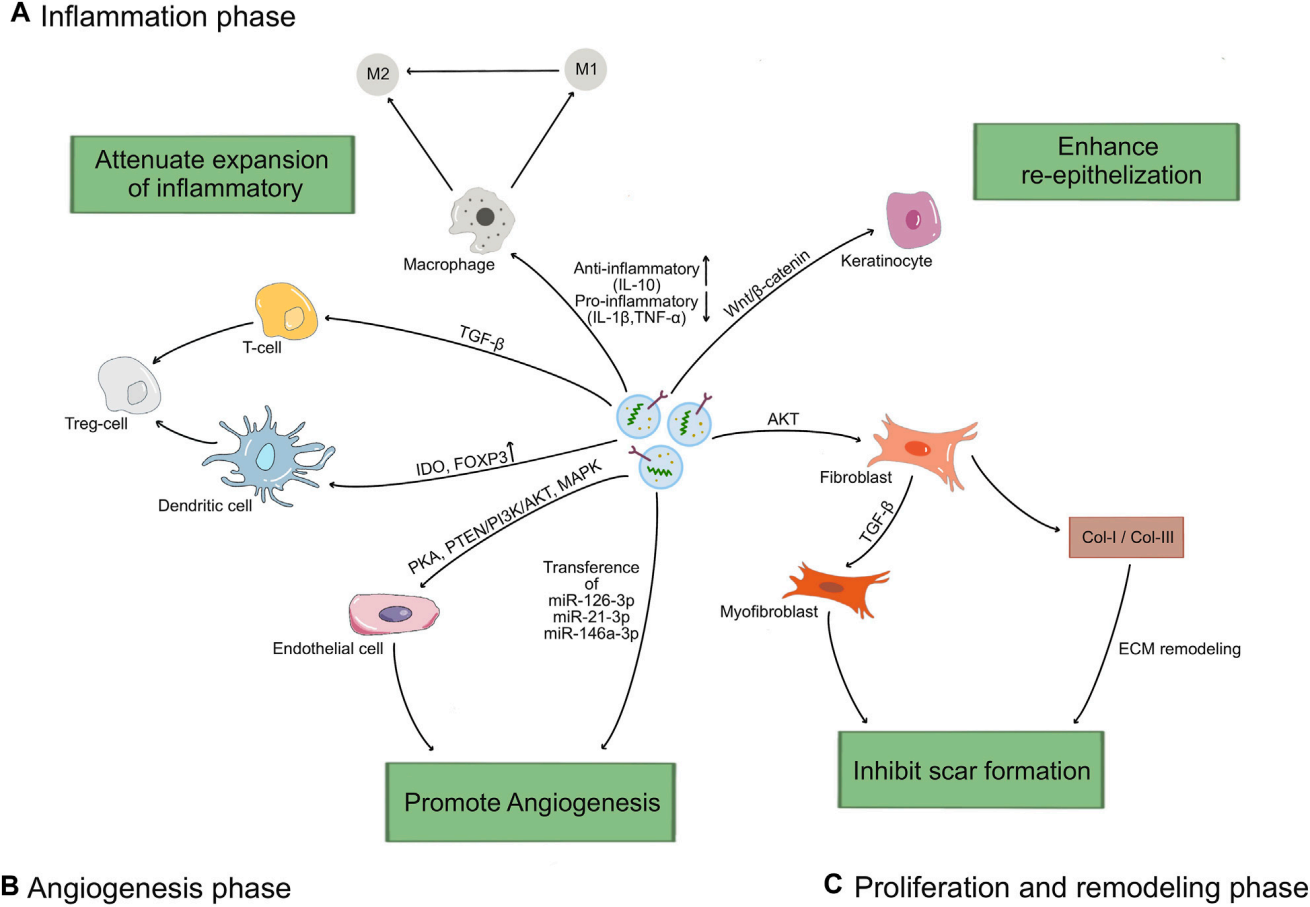
The mechanisms of exosomes in wound healing process. **(A)** Exosomes increase the M2/M1 polarization rate by up-regulating the driver anti-inflammatory cytokines (IL-10) and down-regulating the levels of inflammatory factors (IL-1β, TNF-α), and induce T cells differentiated into Treg cells through TGF-β pathway, upregulation IDO or Foxp3. **(B)** Exosomes stimulate endothelial cells proliferation and migration through activating the PKA pathway or PTEN/PI3K/AKT pathway or MAPK pathway, and transferring miRNAs (miR-126-3p, miR-21-3p, miR-146a-3p) to promote angiogenesis. **(C)** Exosomes promote the proliferation and migration of keratinocytes via Wnt/β-catenin pathway to enhance re-epithelization, and fibroblasts via AKT pathway to promote ECM synthesis and remodel. Lately, exosomes regulate the differentiation of fibroblasts to myofibroblasts, and the ratio of collagen I to collagen III to reduce scar formation.

In various phases of wound healing process, exosomes regulate the function of keratinocytes, fibroblasts, macrophages and endothelial cells, laying a better foundation for reducing inflammation, forming subsequent angiogenesis, accelerating the proliferation phase, and aiding in tissue remodeling (Figure 3). In the subsequent section, we will elaborate on the impact of exosomes on wound healing (Table 1).

**TABLE 1:** The mechanisms of exosomes in wound healing process.

Wound healing phase	Source	Cargo	Model	Function	Reference
Inflammatory	ADSCs	miR-223	Mouse model of cardiotoxin-induced muscle injury	Enhance the expression of anti-inflammatory factor (IL-10) and reduce the expression of pro-inflammatory factor (IL-6)	Lo Sicco et al. (2017)
	UCMSCs	miR-181c	Rat skin burn model	DownregulateTLR4, NF-κB/P65 and p-P65 protein expression, preventing the release of inflammatory factors such as IL-1β and TNF-α	Li et al. (2016)
	BMSCs	miR-361-5p	Osteoarthritic rat	Inhibit NF - κ B signaling pathway, reducing the level of pro-inflammatory factors (IL-6 and TNF -α)	Harrell et al. (2019)
Angiogenesis	UMSCs	wnt-4	Rat skin burn model	Active β-catenin to promote angiogenesis	Zhang et al. (2015)
	ADSCs	VEGF	Mouse model of fat grafts	Enhance angiogenesis through VEGF/VEGF-R	Han et al. (2020)
	UCBs	miR-21-3p	Full-thickness skin wounds in mice	Enhance the angiogenic activities of endothelial cells	Hu et al. (2018)
	ADSCs	miR-146a	Skin-defect mouse model	Increase the CD31 expression to promote angiogenesis	Chen et al. (2023)
	SMSCs	miR-126	Diabetic rat model	Promote migration and tube formation of HMEC-1	Tao et al. (2017)
	BMSCs	miR-21-5p	Rat skin wound	Promote proliferation, migration, and tube formation in HUVECs and HSFs	Wu et al. (2020a)
Proliferation	UCMSCs	Wnt/β-catenin	Rat skin wound	Enhance wound closure and inhibit acute heat stress-induced skin cell apoptosis via activation of AKT pathway	Zhang et al. (2015)
	ADSC	miR-146a-3p	Mouse	Promote fibroblast proliferation and migration by inducing expression of serpin family H members and p-ERK2	Hu et al. (2018)
	SMSCs	miR-126-3p	Diabetic rat model	Stimulate the proliferation of human dermal fibroblasts and human dermal microvascular endothelial cells (HMEC-1) in a dose-dependent manner	Tao et al. (2017)
	ESCs	miR-200a	D-galactose-induced aging mice	Accelerate impaired proliferative migration and angiogenesis by down-regulating Keap1 regulation of the activated nuclear factor erythropoietin 2-related factor	Chen et al. (2019)
	ADSC	miR-124 miR-19b lncRNA-H19	Normal human subcutaneous adipose tissue	Accelerate the proliferation and migration of human dermal fibroblasts	He et al. (2020)
Remodeling	ADSCs	MALAT1 lncRNA	HaCaT and HDF cells impaired by H_2_O_2_	Target miR-124 and active the Wnt/β-catenin pathway Promote cell proliferation, migration and inhibit cell apoptosis	He et al. (2020)
		miR-192-5p	skin-defect mice model	Target IL-17RA to regulate Smad pathway and decrease the pro-fibrotic proteins levels and attenuate hypertrophic scar	Li et al. (2021)
	UMSCs	miR-21 miR-23a miR-125b miR-145	skin-defect mouse model	Inhibit the TGF-β/SMAD2 Pathway, reduce scar formation and myofibroblast accumulation	Fang et al. (2016)

### 4.1 Wound inflammatory

Injury leads to immediate activation of clotting cascade to initiated the recruitment of inflammatory cells. It has been verified that the increased pro-inflammatory cellular infiltrates, largely composed of polymorphonuclear neutrophils (PMNs), macrophages and lymphocytes, contribute to delayed wound healing. More evidences demonstrated that MSC-exos exerted an immunosuppression effect by interacting with immune cells or regulating the ratio of pro-inflammatory and anti-inflammatory factors (Whiteside, 2018; Qian et al., 2021). For instance, MSC-exos can increase the M2/M1 polarization rate by modulating the levels of inflammatory factors, such as up-regulating the driver anti-inflammatory cytokine (IL-10) and down-regulating the levels of inflammatory factors (IL-1β, TNF-α) (Curtale et al., 2013; Yang et al., 2015; Li et al., 2016; Lo Sicco et al., 2017; Tao et al., 2021). Exosomes can induce differentiation of T cells and increase the transformation to anti-inflammatory phenotypes, such as regulatory T (Treg) cells, through up-regulating IDO, Foxp3 (Chen et al., 2016; Harrell et al., 2019; Heo et al., 2019; Zhou et al., 2021). These studies indicate that exosomes can suppress the occurrence of excessive inflammation and the adverse effects of inflammation during wound healing process.

### 4.2 Wound angiogenesis

Angiogenesis plays a key role in wound healing. This process mainly includes the proliferation, migration and tube formation of vascular endothelial cells (Demidova-Rice et al., 2012). New blood vessels carry oxygen, nutrients, and various growth factors to maintain the survival, proliferation, and differentiation ability of new tissues (Baru et al., 2021). Exosomes contain a variety of growth factors (VEGF, BFGF, and HGF), cytokines and chemokines, which promote endothelial cell proliferation and migration indirectly promote angiogenesis (Han et al., 2020). HUVECs-exos upregulated the expression of endogenous VEGF upon activation of PKA signaling, as well as the pro-angiogenic genes Angpt1 and Flk1 (Xue et al., 2018). Delivery of HGF by exosomes can maintain vascular stability and promote neovascularization through activating the PTEN/PI3K/Akt and MAPK pathways (Oliveira et al., 2018; Zhang et al., 2018; Yan et al., 2021; Yang et al., 2021; Zhao et al., 2022). In addition, a variety of miRNAs contained in exosomes can also promote endothelial cell proliferation and migration. For instance, MSCs-exos include miR-126-3p, miR-21-3p, miR-146a-3p have the ability to increase the proliferation and migration of endothelial cells via PI3K/AKT and ERK signaling pathways, respectively (Tao et al., 2017; Hu et al., 2018; Wu et al., 2020b; Chen G. Q. et al., 2021). Taken together, these findings indicate that exosomes have great potential on angiogenesis to promote wound healing.

### 4.3 Wound proliferation

The wound proliferative phase mainly focuses on cell proliferation, re-epithelialization and synthesis of matrix proteins to form the granulation tissue, providing sufficient oxygen and nutrients for wound healing and better regeneration of damaged tissue (Eming et al., 2014; Bian et al., 2022). Epithelial cells at the wound edge proliferate and migrate towards the wound center, promoting re-epithelialization and closure of the wound edge. UMSCs-exos were revealed to inhibit HaCaT cell apoptosis by activating the Wnt/β-catenin signaling pathway and promote cell proliferation and re-epithelialization in a rat model of deep second-degree burns (Zhang et al., 2015; Ma et al., 2019). Exosomes from different stem cells have been shown to promote migration and proliferation of fibroblasts. ADSCs-exos can be internalized by fibroblasts and stimulate fibroblast proliferation, migration, and collagen synthesis in a dose-dependent manner (An et al., 2021). The granulation tissues, mainly composed of fibronectin, replaces the provisional extracellular matrix (ECM) during the proliferative phase and creates a scaffold for early deposition of type III collagen and later type I collagen synthesis (Diegelmann and Evans, 2004). Han et al. (2021) revealed that exosomes derived from autologous dermal fibroblasts promote collagen deposition to stimulate diabetic cutaneous wound healing by the Akt/β-catenin pathway. Zhao et al. (2021) showed that ADSCs-exos also accelerate wound healing by promoting collagen synthesis and deposition. In summary, exosomes from different stem cell sources can have a promoting effect on granulation tissue expansion, re-epithelialization, and matrix protein synthesis.

### 4.4 Wound remodeling

Wound remodeling phase mainly includes secretion of extracellular matrix (ECM), collagen remodeling, and the proliferation of neoplastic granulation tissue to cover the damaged area. During the reorganization and remodeling of collagen fibers in damaged skin, exosomes derived from ADSCs contain lncRNA MALAT1 and other factors that stimulate the migration and angiogenesis of fibroblasts, which promote the remodeling of collagen fibers (He et al., 2020). Exosomes also modulate TGF-β levels, causing myofibroblasts to appear transiently, promoting wound contraction and collagen fiber reorganization (Fang et al., 2016). But overproliferated fibroblasts and over-deposited collagen may result in scar formation. In contrast, ADSC-exos reduced collagen deposition after injection into injured tissues *in vivo*, increased the ratio of collagen III to collagen I, and inhibited ECM over-deposition by secreting Matrix metalloproteinase-1 (MMP-1), attenuating scar formation (Hu et al., 2016; Wang et al., 2017; Li et al., 2021). These studies suggest that exosomes from different stem cell sources play a crucial role in promoting collagen remodeling and ameliorating scarring, and are expected to provide new therapeutic strategies for wound treatment.

## 5 The role of exosome in wound healing process

**FIGURE 4:**
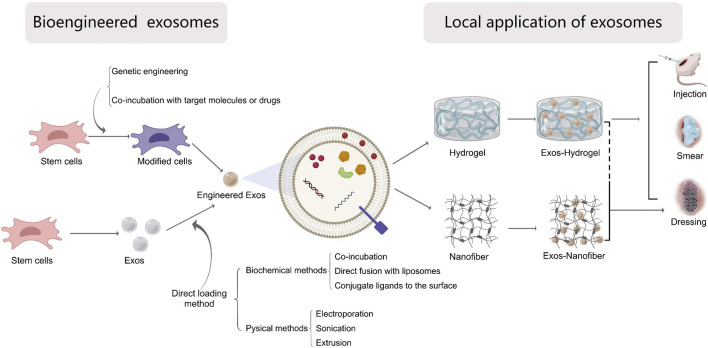
The local application of bio-engineered exosomes into wound healing. Exosomes can be modified by the following ways: 1) Direct exosomes engineering and 2) parental cell-based exosomes engineering. The methods of direct modification are divided into chemical methods including co-incubation, direct fusion with liposomes packaging targets molecules and conjugate ligands to the exosomal surface, and physical methods including electroporation, sonication and extrusion. The parental cell-based exosomes engineering can be achieved by genetic engineered cells or changing the cultural environment of cells or incubating with desired molecules. Biomaterials-based exosomal delivery systems for local application depends on different forms, including hydrogels, nanofiber materials or the combination of both via injection, smearing or wound dressings.

Through the above aspects, exosomes were a promising approach for treating wound healing. However, its clinical efficacy still depends on many other factors, such as specific target capability, delivery methods and high concentration stability. In this part, we will summarize and discuss how to engineer exosomes, optimize delivery methods and modify dosage and frequency (Figure 4).

### 5.1 Bioengineering modified exosomes

To improve the effective application of exosomes for accelerating wound healing, bioengineering techniques have been incorporated by researchers into exosome-based therapy to enhance their targeting efficacy, loading efficiency and stability. Currently engineering strategies mainly fall into two categories: direct exosome engineering and parental cell-based exosome engineering (Lu et al., 2022). These methods result in different loading efficiency and stability of agents with exosomes.

#### 5.1.1 Direct exosome engineering

The exosomes usually work as a carrier, target substrates can be directly delivered into exosomes through biochemical or physical methods.

##### 5.1.1.1 Biochemical methods

The biochemical methods to modify exosomes is an easy, fast but high effective method. Some small-molecule drugs and RNAs were incorporated into exosomes via directly co-incubating, which interact with the lipid layers of membrane (Zhuang et al., 2022). This method is limited to the low loading efficiency, depending on the gradient concentration and the hydrophobicity of drugs (Sadeghi et al., 2023). A surfactant molecular saponin could increase membrane permeabilization, improving the loading efficiency (Fuhrmann et al., 2015; Johnsen et al., 2016). However, there are concerns regarding the concentration of saponin, especially when the exosomes are used *in vivo* (Johnsen et al., 2016).

Another method is direct modification through membrane fusion with liposomes embedded with target molecules. Matsuoka T et al. created HER2-containing exosomes with liposomes via freeze-thaw method (Matsuoka and Yashiro, 2015). This strategy not only improves the characteristics and stability of exosomes, but also reduce the immunogenicity.

Another strategy is to conjugate functional ligands to the surface of exosomes using chemical methods, such as click chemistry or azide-alkyne cycloaddition (Villata et al., 2020). For instance, the ligand cyclo (Arg-Gly-Asp-D-Tyr-Lys) peptide [c (RGDyK)], targeting at integrin α_v_β_3_, were conjugated to modify exosomes (Arosio and Casagrande, 2016; Khan et al., 2016). This conjugation reactions were mildly on exosomes structure or function, maintaining the size of exosomes (Smyth et al., 2014). Based on this strategy, metabolic glycoengineering technique is created to track the localization of the therapeutic cells through exosomes. In above, biochemical modification to the exosome surface can determine cell-type targeting specificity, overcoming off-target effects. However, this method may cause exosome aggregation or surface protein inactivation (Mondal et al., 2023). And it needs attention that temperature, salt concentration or pressure used may result in surface protein denaturation, membrane rupture or excessive osmotic pressure.

##### 5.1.1.2 Physical methods

Electroporation is a promising physical strategy for large compounds which is difficult to encapsulate in exosomes. Electroporation can create small temporary pores on the exosome membrane by disturbing the phospholipid bilayer through an electrical field (Luan et al., 2017). For instance, loading miR-21-5p into ADSCs-derived exosomes by electroporation methods demonstrated excellent effects on skin wound healing through accelerating re-epithelialization, angiogenesis and collagen remodeling (Lv et al., 2020). Yan et al. successfully encapsulated miR-31-5p mimic into milk-derived exosomes, and then showed that it could enhance wound healing by promoting angiogenesis *in vivo* (Yan et al., 2022a).

Sonication is also widely used method for exosome engineering. For this method, drugs are diffused into the exosomes through compromising the exosome membrane integrity by the mechanical shear stress from the sonicator probe. Kim et al. (2016) observed that the membrane integrity of the exosomes is recovered within an hour. In certain instances, drugs not only are encapsulated inside the exosomes but also adhered to the membrane’s outer layers, causing a bulk of drugs firstly burst and release and the following slow release. Generally, the sonication method has more higher loading efficiency than co-incubation and electroporation (Luan et al., 2017).

Extrusion is an another method to load exogenous cargos to exosomes by harsh mechanical force. In contrast to electroporation, the exosome membrane is disrupted and then followed by drugs passing through (Khongkow et al., 2019; Van Deun et al., 2020; Hajipour et al., 2021). According to previous studies, extrusion has some important shortcomings, including low throughout, low loading efficiency and the disruption of the membrane stability resulting in other undesirable side effects (Fuhrmann et al., 2015; Zhao et al., 2023).

#### 5.1.2 Parental cell-based exosome engineering

The parental cell-based exosome engineering aims to produce specifically labeled exosomes or improve the yield of exosomes without any change in exosome structures. The genetic engineering of parental cells was a convenient and stable method. The donor cells are modified via lentivirus or specific mRNAs by inserting the desired target-coding sequence, and then the exosomes carrying target cargoes were isolated and applied for wound healing (Golchin et al., 2022). Exosomes produced from Nrf2-overexpressed ADSCs could lower the inflammation level, enhance the formation of granulation tissue formation and promote the growth factor expression (Li et al., 2018). Exosomes derived from circular RNAs (circRNAs) mmu_circ_0000250 modified ADSCs promoted wound healing by absorption of miR-138-3p and upregulation of SIRT1.

Another method to change the cargoes in exosomes is realized by changing the cultural environment of donor cells or incubating with desired molecules. This method focuses on the preconditioning of parental cells, mainly include hypoxic, cytokine and chemical preconditioning (Lu et al., 2023). For example, atorvastatin-pretreated MSCs promote diabetic wound healing by enhancing angiogenesis through AKT/eNOS pathway (Yu et al., 2020). Exosomes derived from ADSCs cultured under hypoxia condition accelerate wound healing via activating PI3K/Akt pathway (Wang et al., 2021). Compared with genetic engineering of parental cells, this method is relatively simple, but the loading efficiency is low and cytotoxic (Antimisiaris et al., 2018).

In summary, no matter direct bioengineering modified exosomes or parental cell-based exosomes engineering, they in a certain extent showed benefits to the wound healing. However, the contents of exosomes were diverse and complex. The alteration of a single components may cause other unexpected changes, resulting in some safety concerns.

### 5.2 The delivery methods of exosomes to wound healing

From above aspects, engineered exosomes might be a promising approach for accelerating wound healing. But exosomes not only are rapidly degraded from the systemic circulation, but also are easier to aggregate over time, which limits the clinical application. Therefore, to improve the therapeutic effect of exosomes, the combination of exosomes and biomaterials were applied to deliver exosomes, achieving a controlled way to release the exosomes for long time, which can be organized into local application combined with scaffold materials and systemic application by injection.

#### 5.2.1 Local application

##### 5.2.1.1 Direct local injection

Materials loaded with exosomes delivery methods for skin wounds includes local injection and wound dressing. Most studies applied hydrogels by subcutaneous injection onto the wound bed, or around the wounds, or combination of both methods. Some studies delivered hydrogels by intradermal injection around the wound beds, directly interacting with cells in the dermis. To perforate through subcutaneous tissue microneedles (MNs) have progressively attracted more attention. However, the injection method may bring pain to the patients, and the dosage or frequency are unavoidable and intractable issues. In addition, free exosomes injected are usually lapsed rapidly. Another delivery method, biomedical dressings have been discussed extensively in the application of exosomes in wound healing therapy. To maintain a desirable moist environment and exosomes biological activity, hydrogel was widely used and studied. However, the small pore diameter, low porosity and friability limited its application. Therefore, the combination of fiber and hydrogel will become the major trend of wound dressing in the future.

##### 5.2.1.2 Biomaterials-based delivery

Nowadays, biomaterials-based exosome delivery systems for local application depends on different forms, including hydrogels, nanofiber materials or the combination of both.

##### 5.2.1.3 Hydrogels in exosome-based therapy

Hydrogel are three-dimensional hydrophilic polymer networks by cross-linking to form matrices with water. Because of their inherent similarities to extracellular matrix (ECM), good biocompatibility, favorable oxygen transport and high water content, hydrogels are employed in drug delivery systems as desired therapeutic carriers. The following polymers are commonly used to make hydrogels: Natural (alginate, chitosan, gelatin, collagen and so on), or synthetic (polyethylene glycol, PEG), polylactic-co-glycolic acid, PLGA), poly (hydroxyethylmethacrylate, PHEMA), or the combination of both.

The hydrogel encapsulated exosomes through the following three methods: i) Mix the polymer with the exosomes, and then add a crosslinker to form 3D hydrogel *in vitro*, which benefits from optional properties such as mechanical strength and size when applied into the wound healing. Jiang et al. fabricated a matrix metalloproteinase (MMP)-response hydrogel by mixing PEG, maleimide, sulfhydryl and MMP to deliver ADSC-exos into wound sites (Jiang et al., 2022b). ii) The polymers and exosomes are firstly incorporated to the target sites, and followed by the addition of crosslinkers to form gels *in vivo*, which can be delivered by direct injection. Wang et al. (2019) constructed a hydrogel by Pluronic F127, poly-Ɛ-L-lysine and oxidative hyaluronic acid *in vivo* to deliver ADSC-derived exosomes intended for wound healing. For above two methods, the presence of crosslinker may cause cytotoxicity *in vivo*. iii) Immerse the dehydrated porous hydrogels in an exosome-containing solution, called as “breathing” technique. Dehydration of hydrogels can be achieved by lyophilization or solvents. By using this method, Xu et al. (2018) developed a hydrogel containing chitosan and silk fibroin to deliver platelet-rich-plasma-derived exosomes. This method presents less cytotoxicity and slowly releases exosomes.

To prolong the function time and the local concentration of exosomes in the wound bed, the scaffolds could slowly release exosomes depending on specific chemical or physical environmental stimuli, named “smart” hydrogels. A matrix metalloproteinase degradable polyethylene glycol (MMP-PEG) hydrogel was fabricated by Jiang et al. (2022b) to regulate the release of exosomes through reacting with MMP proteins. Zhao et al. (2020) developed a ROS-scavenging hydrogel, composed of polyvinyl alcohol (PVA) of a ROS-responsive linker, N1-(4-boronobenzyl)-N3-(4-boronophenyl)-N1, N1, N3, N3-tetramethylpropane-1, 3-diaminium (TPA) to promote wound closure by decreasing the ROS accumulation in the infective wound environment. The goal of both control and gradual release is to maintain the local exosomes or drugs at suitable concentration depending on the specialized micro-environment.

To assist the therapeutic effect of exosomes, the hydrogels are designed to benefit the wound healing, including increasing oxygen enrichment, accelerating cell proliferation, migration and angiogenesis formation, and improving the antibacterial properties. For example, Liu et al. fabricated a AMSC-exos-loaded β-chitin nanofiber (β-ChNF) hydrogel, which accelerated re-epithelialization and increased collagen expression in the rat full-thickness skin injury model (Liu et al., 2022). Parvaiz et al. loaded exosomes into an oxygen-releasing antioxidant scaffold, polyurethane-calcium peroxide cryogels, which controlled the continuous release of oxygen and exosomes for more than 10 days (Shiekh et al., 2018). Geng et al. (2022) reported a carboxyethyl chitosan-dialdehyde carboxymethyl cellulose (CEC-DCMC) hydrogel, which exhibited remarkable antibacterial characteristics against the further infection of diabetic wounds. Although the loading scaffolds indeed exhibit various excellent characteristics, adding some non-medical components into materials is inevitable, which delay their clinical transformation. So how to balance the efficiency and safety may be further evaluated.

##### 5.2.1.4 Nanofiber materials in exosome-based therapy

The nanomaterials also are an ideal wound dressing considering good biocompatibility, low toxicity, colloidal stability and acceptable biodegradability, which can carry exosomes through the nanoscale properties.

Khalatbari et al. (2022) entrapped the alginate loaded with BMSCs-exos into silk fibroin to make a naturally-based polymers which had reasonable water vapor transfer rates and good swelling properties and excellent biocompatibility, making them suitable for wound dressing. A functional phosphoethanolamine phospholipid-grafted poly-L-lactic acid micro/nanofibers (DSPE-PLLA) was fabricated to carry and retain the slow release of exosomes from AMSCs, releasing the inflammatory response, promoting cell proliferation, angiogenesis and collagen deposition (Li et al., 2022). Compared to hydrogels, the polymers have more mechanical properties, an increase in diameter of scaffolds, which benefits to cell adhesion, proliferation and differentiation. In addition, proper vapor and oxygen permeability more easily balanced to the excretion of wound exudates, reducing the accumulation of bacteria and other infection factors. However, the polymers are generally combined with hydrogels, which are more conductive to the biological viability of exosomes. In addition, nanocomposites with nanomaterial-modified microfluidic channels are expected to achieve high capture rates and high throughput screening of exosomes in the future (Zou et al., 2023).

#### 5.2.2 Systemic application

For systemic application of exosomes to enhance wound healing, we found Han et al. (2022) delivered BMSC-derived exosomal IncRNA KLF3-AS1 via tail vein injection. In addition, Zhou et al. systematically study the effect of different local ADSC-Exos smearing, injection and systemic intravenous administration for non-diabetic wound healing, and results showed that the combination of local smearing and intravenous administration could promote re-epithelialization, improve angiogenesis and collagen synthesis, achieving a fast wound healing process. Wounds associated with metabolic diseases, such as diabetes, may face more other systemic problems. The systemic application of exosomes may play a significant therapeutic role in systemic disease.

## 6 Conclusion and perspectives

Skin wound healing is a complex multi-phase biological process. Stem cells have been verified a promising strategy in regenerative medicine. Exosomes, known as a main bioactive factor of MSC via paracrine activity are shown to have the same therapeutic effects as MSCs. And compared to MSCs, exosomes exhibit lower immune rejection and tumorigenesis risk *in vivo* (Zhang et al., 2014). However, many issues should be improved before applying exosomes into clinic.

Firstly, how to achieve a large-scale acquisition of exosomes from parental cells needs to be improved. At present, the more common separation and purification methods, such as ultracentrifugation, size exclusion chromatography, take a long time and complicated steps, resulting in low yield (Zou et al., 2023). Another challenge during the application is the absence of a standard manufacturing process to ensure the purification and clinical safety, limiting the clinical applications. In addition, storage and transport conditions of exosomes need to be addressed, which is studied by researchers to explore the ideal conditions (Levy et al., 2023).

Secondly, research on exosomes is mainly applied on animal models. Few clinical trials have been conducted to validate the effectiveness of exosomes on wound healing. A randomised clinical trial showed that human adipose stem cell-derived exosomes combined with carbon monoxide and laser treatment of acne significantly reduced scarring and erythema, initially confirming the efficacy and safety of exosomes in the clinic, so more clinical trials are needed in the future (Kwon et al., 2020). Moreover, the heterogeneity of exosomes from various sources and states necessitates further characterization to distinguish between subpopulations with distinct functions before utilized in clinical therapy (van Niel et al., 2018).

To improve the targeted therapeutic effects, exosomes can potentially be modified by directly engineering exosomes or modifying parental cells. However, engineered exosomes may face some of the shortcomings, such as low loading efficiency, the biosecurity of application. So it is necessary to rationally regulate the distribution, clearance and therapeutic efficacy of the cargo within exosomes at the wound bed. And how to maximize the therapeutic effect of engineered exosomes combined with materials and more efficient delivery methods needs to be further explore in future.

In conclusion, this review provides new insights into the role of exosomes in skin wound healing and the modification and delivery of exosomes. However, further research is still needed to explore the relevant mechanisms of exosomes in skin wound progression. And more researches are required to make engineered exosomes more accessible and feasible for clinical applications into wound healing.

## Abbreviations

EVs, extracellular vesicles; MVBs, multivesicular bodies; ILVs, intraluminal vesicles; ESCs, epidermal stem cells; MSCs, mesenchymal stem cells; MSC-exos, mesenchymal stem cell-derived exosomes; ADSCs, adipose-derived stem cells; BMSCs, bone marrow mesenchymal stem cells; HUVECs, human umbilical vein endothelial cells; UCMSCs, umbilical cord blood mesenchymal stem; SMSCs, Synovium - derived mesenchymal stem cells; UCBs, Umbilical cord blood mesenchymal stem cells; UMSCs, Human umbilical cord-derived mesenchymal stem cells.
